# Dynamic correlation between chest temperature entropy and physiological load indicators, and excess post-exercise oxygen consumption during incremental cycling exercise

**DOI:** 10.3389/fphys.2025.1631729

**Published:** 2025-09-10

**Authors:** Songzi Cui, Ning Du, Zhongqian Liu, Chenxi Hu

**Affiliations:** ^1^ Department of Orthopaedics, Fourth Medical Centre of Chinese PLA General Hospital, Beijing, China; ^2^ Department of Chinese Academy of Sport and Health, Beijing Sport University, Beijing, China

**Keywords:** thermal imaging, entropy analysis, incremental exercise protocol, oxygen consumption, blood lactate response

## Abstract

**Objective:**

This study aimed to investigate the dynamic relationship between chest temperature entropy, physiological load indicators, and excess post-exercise oxygen consumption (EPOC) during incremental cycling exercise using high-sampling-rate infrared thermography (IRT).

**Methods:**

Twenty-four healthy young male participants (23.7 ± 3.3 years; 178.6 ± 9.8 cm; 78.5 ± 6.4 kg; 237.8 ± 48.7 min/week training duration; maximal oxygen uptake 44.06 ± 5.9 ml/kg/min; maximal power output 263.8 ± 27.4 W) performed an incremental cycling test starting at 60 W with workload increases of 30 W every 2 minutes until exhaustion. Chest thermography, oxygen consumption (VO₂), blood lactate, and external load were simultaneously measured, and entropy was used to quantify the spatial complexity of temperature distribution.

**Results:**

Significant non-linear positive correlations were found between standardized entropy increase and VO₂ (*R*² = 0.809), blood lactate (*R*² = 0.719), and external load (*R*² = 0.841), while individual-level analyses confirmed strong associations with VO₂ (r = 0.874–0.977), lactate (r = 0.692–0.989), and external load (r = 0.889–0.986) (all p < 0.05). Hierarchical clustering identified three clusters corresponding to low, moderate, and high metabolic load states. During recovery, entropy was significantly associated with EPOC (*R*² = 0.151), though substantial inter-individual variation was observed (r = –0.288 to 0.907).

**Conclusion:**

Chest temperature entropy dynamically reflects exercise-induced metabolic changes and partially explains recovery processes, highlighting its potential as a novel, non-invasive marker for monitoring exercise load and recovery.

## 1 Introduction

Training Load is the quantification of training stimulus, and through systematic and periodic application of load, it induces acute metabolic regulation, neural activation, short-term physiological stress, and long-term functional adaptation (muscle hypertrophy, enhanced cardiovascular function), leading to the systematic improvement of athletic performance (Bourdon et al., 2017; Halson, 2014; Lu et al., 2024). Modern load monitoring systems assess both external and internal loads to construct a mapping relationship between training stimulus and physiological responses (Im et al., 2019). External load monitoring relies on wearable devices such as GPS and accelerometers, to quantify training volume (duration, distance, power) and intensity (speed, acceleration) through physical parameters (Borresen and Lambert, 2009). Power meters quantify mechanical work output as an objective indicator of external load, providing valuable information for monitoring training adaptation and preventing non-functional overreaching (Halson, 2014; Sitko et al., 2020). Internal load monitoring, on the other hand, uses multidimensional data such as blood biomarkers related to creatine kinase, testosterone/cortisol ratio, heart rate variability, and subjective fatigue scales to understand the body’s metabolic state and recovery progress (Halson, 2014).

In practical application, external load monitoring requires the use of sensor devices, which may interfere with the athlete’s freedom of movement. While the gas metabolic measurement system, considered the gold standard for aerobic metabolism (Poole and Jones, 2017), offers high reliability, it is limited by the cost of equipment and restricted application scenarios (Shei et al., 2022). Blood lactate threshold testing, though sensitive to identifying anaerobic thresholds, faces challenges with low time resolution due to discrete sampling and invasive procedures, potentially triggering cortisol stress responses (Faude et al., 2009). Although traditional monitoring technologies are well-established in sports science practice, their invasiveness, high costs, and the lack of localized physiological data may limit their application in dynamic, continuous, and individualized load monitoring.

Infrared thermography (IRT) has emerged as a new tool in sports science research due to its non-invasive nature, mobility, and high spatial-temporal resolution (Costello et al., 2013). IRT captures infrared radiation from the body surface, generating temperature distribution images, and associates changes in signal intensity with muscle micro-damage and local metabolic rate responses during exercise (Masur et al., 2024). Recent studies have shown that IRT effectively captures local temperature changes induced by exercise to detect muscle activity, and the dynamic changes in skin temperature during exercise have become a well-established method to study the relationship with exercise load in sports science (Moreira et al., 2017). Research indicates that during incremental cycling tests, the maximum temperature of the bilateral thighs decreases significantly due to vasoconstriction triggered by increased metabolic demand, while temperature rises during the recovery period (Ludwig et al., 2016). This phenomenon is likely directly related to energy metabolism and blood flow distribution to the skeletal muscles. A similar cooling trend is also evident in resistance training, where the skin temperature (Tsk) in six regions of interest in the lower limbs decreases to varying degrees following 70% 1RM resistance exercise, and the temperature changes in the thighs and knees are negatively correlated with sympathetic nervous system activation (Sillero-Quintana et al., 2022). In aerobic load experiments, skin temperature shows a moderate positive correlation with maximal aerobic capacity (r = 0.6), and a stronger negative correlation with lactate levels (r = −0.7), supporting its potential as a metabolic load quantification indicator and dynamic monitoring tool for exercise load (Akimov et al., 2010). Under specific exercise modes, local skin temperature changes reflect muscle activation patterns and adjustments in blood flow dynamics. The Perpetuini team, through simultaneous collection of surface electromyography (sEMG) signals and infrared thermography data during squat-to-fatigue exercises in ten healthy participants, found significant positive correlations between the temperature features extracted using an improved Gaussian regression model (in the time domain, frequency domain, and non-linear features) and the average rectified value and median frequency of sEMG signals. In a study assessing thermal asymmetry in the body surface temperature distribution of boxers, the temperature of the posterior abdominal and lumbar regions was found to be 0.5 °C and 0.4 °C higher, respectively, compared to the anterior side, while the temperature of the anterior calf and fist joints was higher by 0.4 °C and 0.3 °C, respectively (del Estal et al., 2017). This research suggests that the asymmetry in body surface temperature may be related to the athletes’ movement patterns and specific muscle activation during training and competition. Additionally, a study analyzing Tsk changes in male endurance runners with high and moderate aerobic capacity during a progressive maximal load test found that the high aerobic capacity group had significantly higher Tsk at baseline, 60%, and 70% of maximal load compared to the moderate aerobic capacity group. Peak Tsk was positively correlated with variables such as age, body fat percentage, muscle mass, VO_2_peak, maximum speed, heart rate, and ventilation (Galan-Carracedo et al., 2019).

Current research on skin temperature feature extraction primarily focuses on traditional parameters such as average temperature, extreme values, and temperature difference (Perpetuini et al., 2021), while entropy, as a measure of the complexity of temperature distribution, has not been fully explored. Entropy quantifies the average information content of an event relative to the probability distribution of a random variable, and in infrared thermography it reflects the degree of spatial heterogeneity within a region of interest (ROI) (Verderber et al., 2024). During incremental cycling exercise, chest temperature entropy has been observed to increase progressively with exercise intensity and to show a stronger correlation with pulmonary ventilation than mean temperature measures (Bini et al., 2011). Further studies have confirmed significant positive correlations between chest temperature entropy and both external power output and oxygen uptake during incremental load, highlighting its sensitivity to subtle spatial variations in skin temperature and its ability to capture complex thermal patterns that are not discernible through conventional temperature metrics (Verderber et al., 2024; Hu et al., 2025). One experimental study investigated the effect of local cooling on skin blood flow during hyperemia, using multiscale entropy to assess the efficacy of local cooling in treating reactive hyperemia and the degree of skin temperature ischemia in individuals with spinal cord injuries (Liao et al., 2019). Another study aimed to determine whether changes in skin temperature characteristics could accurately predict the risk of pressure ulcers, observing the entropy and spectral indices of the skin temperature in participants. Although there were no significant differences in the probability of ulcer formation across the multiscale skin temperature measurements, the study provided a new tool for evaluating the regulation of skin temperature and, from another perspective, assessing the distribution of skin temperature and its relationship with metabolic information (Rapp et al., 2009).

The dynamic relationship between entropy in body temperature distribution and oxygen consumption (VO_2_) has not yet been elucidated in current research. Although studies have shown a strong correlation between chest temperature entropy and pulmonary ventilation (Bogomilsky et al., 2022), there is insufficient research on the quantitative relationship between blood lactate threshold and thermal dysregulation. Additionally, the thermal recovery characteristics during the excess post-exercise oxygen consumption (EPOC) phase remain underexplored, and the variability in the relationship between entropy and physiological indicators across individuals is not yet clear. Furthermore, there is a lack of synchronized validation data for temperature entropy in regions of interest from thermal imaging and physiological parameters such as VO_2_ and blood lactate. Therefore, this study utilizes an incremental cycling exercise experiment, employing IRT to collect chest temperature entropy data during exercise, along with simultaneous measurements of VO_2_, power output, and blood lactate. The study analyzes the relationship between temperature entropy and changes in both internal and external load during the incremental power phase and extends EPOC monitoring for 10 min post-exercise. By analyzing the entropy distribution of infrared thermography–derived skin temperature, this study quantifies metabolic and exercise load without interfering with movement, providing a valuable supplement to existing load monitoring methods. We hypothesize that chest temperature entropy derived from infrared thermography is positively associated with both internal and external loads during incremental cycling, and that it can effectively discriminate metabolic load states and characterize recovery dynamics during the EPOC phase.

## 2 Methods

### 2.1 Participants

A total of 24 young male participants were recruited from Beijing Sport University, all meeting the inclusion criteria: no chronic diseases, no lower limb injuries in the past 6 months, and regular participation in physical exercise. The participants had an average age of 23.7 ± 3.3 years, height of 178.54 ± 9.8 cm, weight of 78.5 ± 6.4 kg, weekly exercise duration of 237.8 ± 48.7 min/week, engaging in activities such as cycling, running, and resistance training, VO_2_max of 44.1 ± 5.9 mL/kg/min, and maximal power output of 263.8 ± 27.4 W. This study was conducted in accordance with the Institutional Review Board of Beijing Sport University (Approval No. 2024303H) and was approved by the Ethics Committee. All participants received detailed information regarding the study’s procedures and potential risks, and informed consent was obtained from each participant.

A power analysis indicated that with n = 24, the study had >80% power (α = 0.05, two-tailed) to detect correlations of ρ ≥ 0.55, which covered the majority of the observed associations.

### 2.2 Thermal imaging

Infrared thermography images during exercise were captured using the IRay AT200 F infrared thermal imaging device (IRay Technology Co., CN). The camera lens has a focal length of 3.2 mm, a resolution of 256 × 192, image frequency of 25Hz/30Hz, thermal sensitivity of 60mk, and a wavelength range of 8–14 μm. The temperature range is from 0 °C to 60 °C, with an accuracy of ±0.5 °C, and the imaging distance is between 0.5m and 3 m. The field of view is 56 ° × 42.2 ° and the emissivity is 0.98. The thermal image captured corresponds to the current frame collected by the temperature measurement thermal imager. During the tests, the laboratory temperature was controlled at 23 °C with a humidity level of 52% ± 2.1% to optimize the performance of the infrared thermography device. Indoor airflow was minimized to reduce the environmental impact on skin temperature. Participants were instructed to expose their torso skin throughout the cycling session while maintaining an upright sitting posture. The imaging area was the chest, and the infrared thermal imager was fixed on a stable support (80 cm from the body), with the temperature sensor angled toward the specific region of interest (ROI). After the device was powered on, it was left to stabilize for 10 min to complete thermal adaptation. During cycling, thermal images were captured every 6 s. In the pre-exercise resting phase, images were collected every minute, and in the 10-min recovery phase, images were captured every 6 s. To eliminate the influence of motion artifacts in the thermal images, the average entropy for the last minute of each workload phase and for every minute of the recovery phase was extracted as the entropy feature for statistical analysis. A total of at least 9,000 thermal images were collected throughout the experiment.

### 2.3 Exercise protocol

Prior to testing, the MONARK 893E ergometer was individually adjusted for each participant, including saddle height, fore-aft position, and handlebar reach, based on anthropometric measurements and the participant’s preferred cycling posture. Saddle height was set according to leg length to ensure an appropriate knee angle at full extension. All participants used the original saddle supplied with the MONARK 893E ergometer to maintain consistency, as saddle design and positioning can substantially influence rider comfort and cycling performance (Bini et al., 2011; Vicari et al., 2023). Participants performed a graded cycling protocol using a MONARK 893E ergometer, beginning with 3 min of seated rest without prior warm-up. The test began at a baseline workload of 60 W, followed by stepwise 30 W increments every 2 min until volitional exhaustion. Standardized verbal encouragement was provided at fixed intervals throughout the test to maintain participants’ motivation. Continuous heart rate monitoring was conducted using a Polar Verity Sense arm-worn sensor (Polar Verity Sense, Finland, Polar Electro Oy). Oxygen uptake (VO_2_) and pulmonary ventilation were measured in real time using the METALYZER® 3B system (Cortex Biophysik GmbH, Germany) and analyzed with MetaSoft Studio software.

**FIGURE 1:**
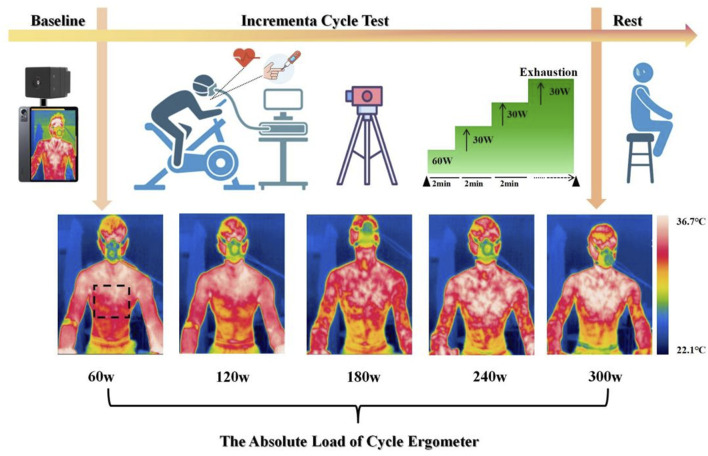
Test process for measuring incremental cycling

During the final 30 s of each workload phase, capillary blood samples were collected from the right medial fingertip using sterile disposable lancets (BD Microtainer, BD, United States). Blood lactate concentrations were measured immediately using the Lactate Scout 4 analyzer (EKF Diagnostics, Cardiff, United Kingdom), which requires 0.2 μL of whole blood and provides results within 12 s using pre-calibrated single-use biosensors (LS4-SM01) (Figure 1).

### 2.4 Feature extraction

Thermal imaging data were securely transmitted via wireless protocols to a dedicated secure server with encrypted storage protocols. Following comprehensive data acquisition, a standardized post-processing pipeline was implemented: manual segmentation of thermal images into predefined anatomical regions of interest (ROIs) was conducted based on standardized anatomical landmarks and spatial calibration protocols, followed by exportation of two-dimensional temperature distribution matrices for each ROI. To ensure spatial consistency, the ROI boundaries for matrix extraction were maintained across all participants using predefined coordinate alignment algorithms.

A multidimensional thermal response dataset was constructed by aggregating temperature matrices across all participants under varying loading conditions, subsequently subjected to dimensionality reduction via feature extraction. Each ROI-specific temperature matrix was processed within the statistical computing environment (R version 4.2.1; R Foundation for Statistical Computing, Vienna, Austria) under the following protocol: raw temperature values were quantized to a resolution of 0.1 °C to mitigate instrumentation noise and normalized within each ROI by subtracting the minimum temperature value. The probability distribution required for entropy computation was then derived from the frequency distribution of these normalized temperature values. Shannon entropy was directly calculated without applying additional filtering or smoothing, in order to preserve subtle local thermal variations that might otherwise be obscured. Entropy-based features were extracted using the Shannon entropy formula (Bogomilsky et al., 2022):

$$H\left(x\right)=-\sum_{k=1}^{n}\;p\left(x_{k}\right)\log_{2}p\left(x_{k}\right)$$ (1)

The entropy value for each ROI was calculated using Equation 1, where p (x_k_) denotes the probability of the k -th temperature value.

### 2.5 Statistical analysis

In this study, all entropy calculations and temperature-related mean analyses were performed using R software (version 4.2.1, R Development Core Team, Vienna, Austria). Data visualization and statistical analyses were conducted using GraphPad Prism (version 8.0, San Diego, CA, United States) and MATLAB 2022a (MathWorks, Natick, MA, United States). Data are presented as means (M) and standard deviations (SD). To evaluate the relationships among temperature entropy, oxygen uptake (VO_2_), blood lactate, and exercise load (power), Spearman’s rank correlation analysis was exclusively employed, as some variables were not normally distributed at the individual level. Prior to analysis, all entropy, VO_2_, blood lactate, and exercise load data were standardized using Z-scores.

Subsequently, hierarchical clustering was applied to the standardized data, with the optimal cluster number determined as 3 based on the highest silhouette score. Statistical significance was determined based on p-values, with differences considered significant at p < 0.05. In addition, we established mathematical equations between standardized entropy and oxygen uptake, as well as between standardized entropy and blood lactate through polynomial fitting, in order to quantify the relationships among these variables.

## 3 Results

### 3.1 Correlation analysis between chest temperature entropy and oxygen uptake

**FIGURE 2:**
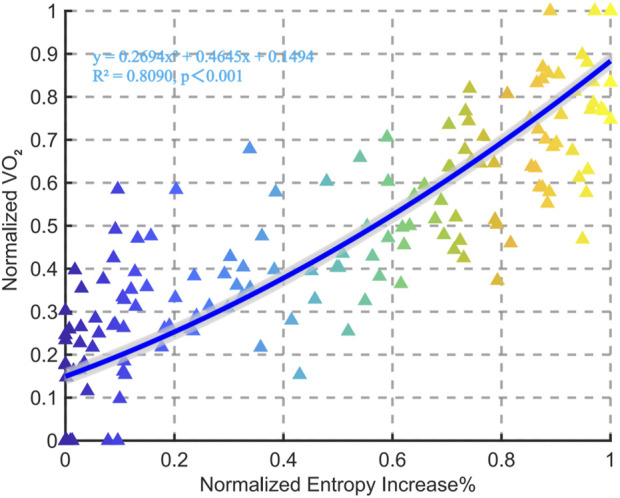
Relationships between normalized entropy increase% and VO_2_ for all data points in the group.

This study performed a polynomial fitting between normalized entropy increase percentage and normalized Vo_2_, revealing a significant positive correlation between the two variables (*R* ^2^ = 0.8090, p < 0.001). Specifically, the increase in entropy is associated with an elevation in blood lactate levels, following a quadratic relationship represented by the equation (y = 0.2694x^2^ + 0.4645x + 0.1494) (Figure 2).

**FIGURE 3:**
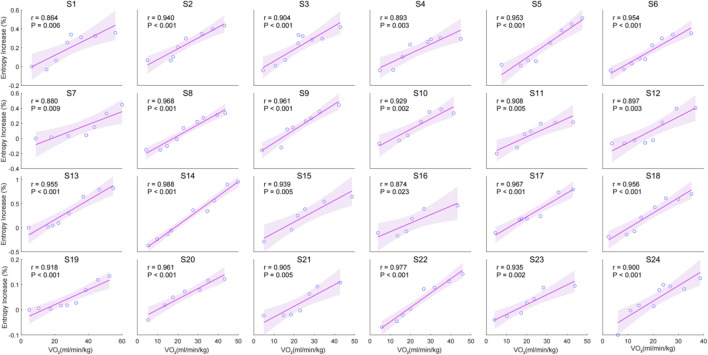
Individual Correlation between entropy increase percentage and VO_2_ for 24 individuals.

The individual correlation analysis indicates a significant positive correlation between the entropy increase percentage and VO_2_ during incremental cycling. The correlation coefficients (r) for all individuals ranged from (r = 0.874) to (r = 0.977), with all correlations being statistically significant (p < 0.05) (Figure 3).

### 3.2 Correlation analysis between chest temperature entropy and blood lactate

**FIGURE 4:**
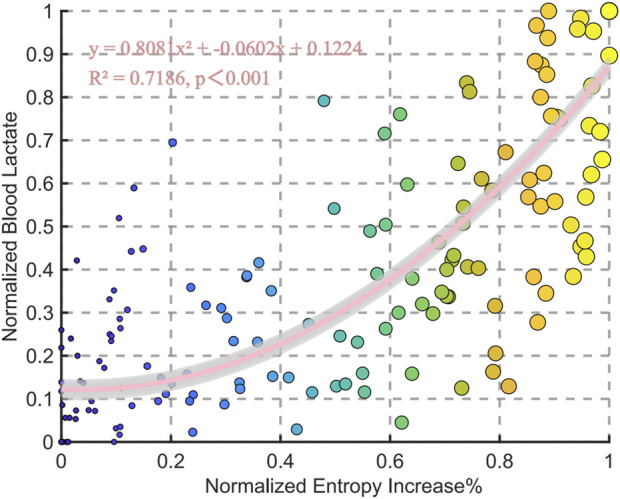
Relationships between normalized entropy increase% and blood lactate for all data points in the group.

This study performed a polynomial fitting analysis between normalized entropy increase percentage and normalized blood lactate, revealing a significant positive correlation between the two variables (*R* ^2^ = 0.7186, p < 0.001). The fitting equation is (y = 0.8081x^2^ - 0.0602x + 0.1224), indicating a strong correlation between entropy increase and blood lactate (Figure 4).

**FIGURE 5:**
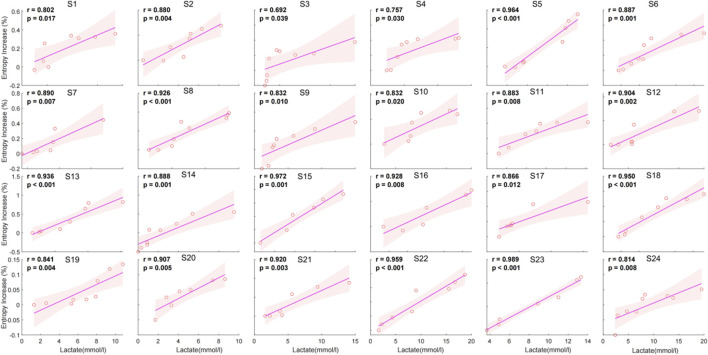
Individual correlation between entropy increase percentage and lactate for 24 individuals.

The individual correlation analysis shows a significant positive relationship between entropy increase percentage and lactate levels across all subjects. The correlation coefficients (r) range from 0.692 to 0.989, with all values achieving statistical significance (p < 0.05) (Figure 5).

### 3.3 Correlation analysis of chest temperature entropy and external load

**FIGURE 6:**
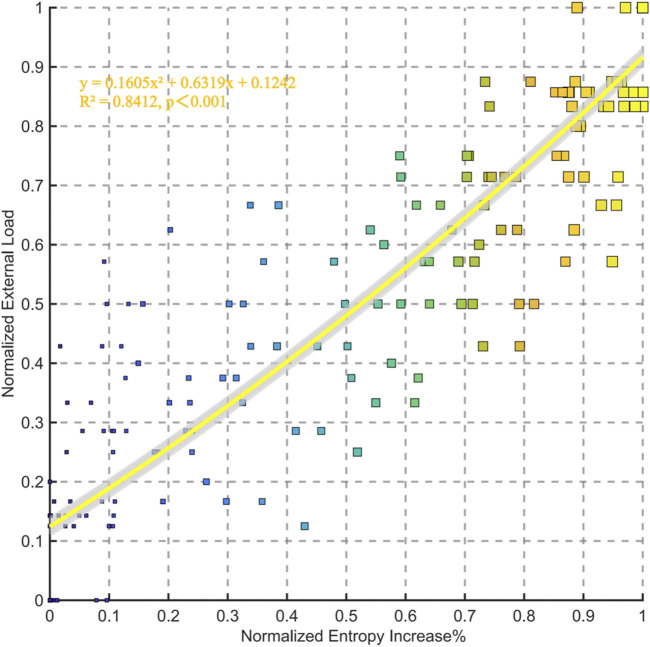
Relationships between normalized entropy increase% and external load for all data points in the group.

The polynomial fitting analysis between normalized entropy increase percentage and normalized external load reveals a significant positive correlation (*R* ^2^ = 0.8412, p < 0.001). The fitting equation is (y = 0.1605x^2^ + 6.6319x + 0.1242), indicating a strong quadratic relationship between entropy increase and external load. As the entropy increase percentage rises, the normalized external load also increases, demonstrating their robust association (Figure 6).

**FIGURE 7:**
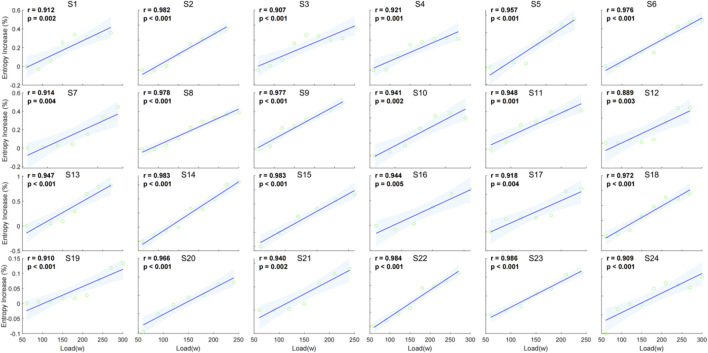
Individual correlation between entropy increase percentage and external load (watt) for 24 individuals.

The individual correlation analysis indicates a strong positive relationship between entropy increase percentage and external load across all subjects. The correlation coefficients (r) range from 0.889 to 0.986, with all values being statistically significant (p < 0.05) (Figure 7).

### 3.4 Correlation analysis of chest temperature entropy and EPOC

**FIGURE 8:**
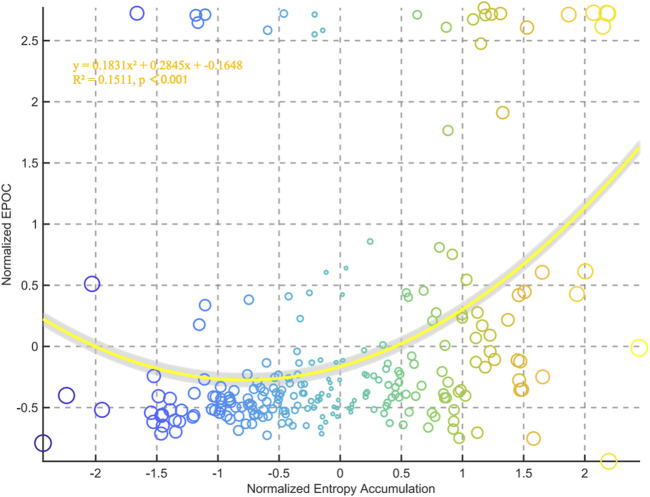
Relationships between normalized entropy accumulation and external EPOC for all data points in the group.

The correlation analysis between chest temperature entropy accumulation and EPOC demonstrated a significant positive association. Polynomial fitting analysis generated the quadratic model y = 0.1831x^2^ + 0.2845x–0.1648 (*R* ^2^ = 0.1511, p < 0.001), statistically validating the proportional relationship between entropy accumulation and EPOC magnitude (Figure 8).

**FIGURE 9:**
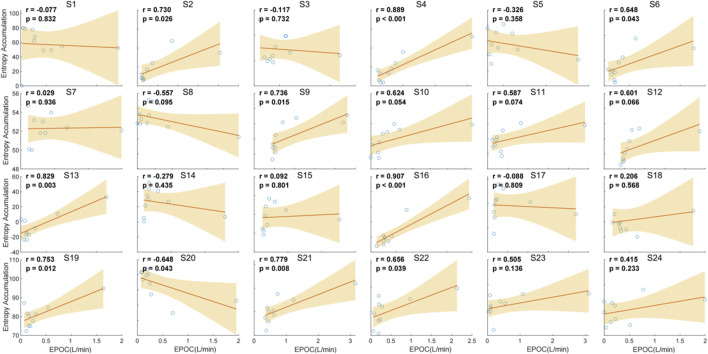
Individual correlation between entropy accumulation and EPOC (L/min) for 24 individuals.

The correlation analysis between chest temperature entropy accumulation and EPOC demonstrates variable relationships across individuals. Significant positive correlations were observed in several subjects, with the strongest correlations found in S4 (r = 0.889, p < 0.001), S11 (r = 0.889, p < 0.001), and S16 (r = 0.907, p < 0.001). Conversely, weaker or non-significant correlations were noted in other subjects, such as S1 (r = 0.077, p = 0.832), S3 (r = −0.117, p = 0.732), and S17 (r = −0.288, p = 0.809). These findings suggest that the relationship between entropy accumulation and EPOC is not consistent across individuals, highlighting individual variability in this physiological response (Figure 9).

### 3.5 Three-dimensional cluster analysis based on chest temperature entropy, oxygen uptake and blood lactate

**FIGURE 10:**
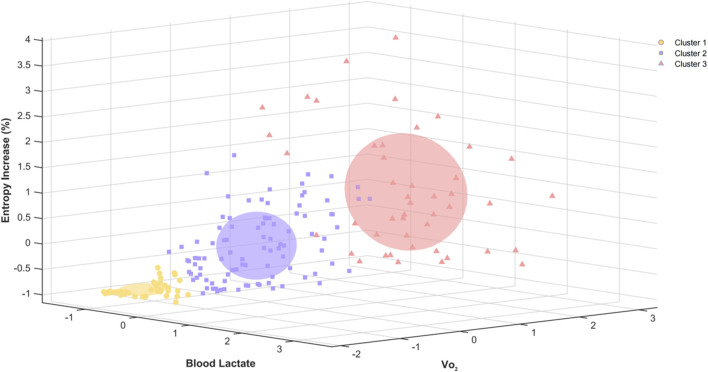
Three-dimensional hierarchical clustering of entropy increase percentage, blood lactate, and VO_2_ based on Z-score standardization.

The hierarchical clustering analysis, based on Z-score standardized entropy increase percentage, blood lactate, and VO_2_, identifies three clusters. Cluster 1 shows low blood lactate, VO_2_, and negative entropy increase. Cluster 2 represents a moderate metabolic state with moderate blood lactate, VO_2_, and positive entropy increase. Cluster 3 reflects a high metabolic demand with high blood lactate, VO_2_, and entropy increase (Figure 10).

## 4 Disscusion

This study reveals the value of chest temperature entropy in monitoring metabolic responses during incremental exercise. By analyzing the multidimensional physiological responses of 24 participants during incremental cycling, we established for the first time a quantitative relationship between the temperature entropy parameter and the energy metabolism system during and after incremental exercise. The findings show that the standardized percentage increase in chest temperature entropy has a significant non-linear positive correlation with VO_2_, blood lactate concentration, and external load. The primary aim was to evaluate the feasibility of infrared thermography–based chest temperature entropy analysis for assessing both internal and external exercise load, as well as characterizing recovery dynamics in the EPOC phase; the findings supported this hypothesis. Three-dimensional hierarchical clustering analysis grouped blood lactate, oxygen uptake, and chest temperature entropy into three clusters, corresponding to low, moderate, and high metabolic load states. The relationship between temperature entropy and EPOC exhibited considerable individual heterogeneity.

The strong positive correlation between chest temperature entropy and oxygen uptake (VO_2_) suggests that an increase in metabolic demand is accompanied by a rise in the disorder of the thermodynamic system of body surface temperature. The increase in entropy serves as a quantitative description of the surface thermal radiation patterns of skin temperature during exercise. During exercise, external load increases muscle work intensity, requiring more oxygen consumption and heat production by the body. Metabolic heat is regulated through superficial vasodilation (Stone et al., 2025; Rosbrook et al., 2024). Research shows that metabolic heat production during endurance exercise can increase by 10–20 times compared to resting, and this increase can persist for several hours (Lim et al., 2008). These studies suggest that thermoregulation ability reflects muscle work and related metabolic information during exercise, and this study further demonstrates that the entropy of body surface chest temperature is strongly positively correlated with oxygen uptake levels both overall and individually.

The difference between this study and previous research on skin temperature and blood lactate lies in the introduction of the concept of thermodynamic entropy. For the first time, this study verifies the strong correlation between blood lactate and chest entropy during exercise, further revealing the regulatory role of anaerobic metabolism in thermodynamics. Adamczyk et al. measured the static lower limb thermal imaging and corresponding lactate collection before and 30 min after completion of a vertical jump exercise in 16 untrained men. They found a weak negative correlation between the two (r = −0.29) (Adamczyk et al., 2014). Although skin temperature and blood lactate in the quadriceps region were reported to be positively correlated (r = 0.69), the intermittent sprint pattern resulted in a temporal misalignment between temperature and lactate sampling, which may have confounded the thermal metabolic signals of exercise stress and recovery (Temfemo et al., 2011). Recent studies have shown a moderate positive correlation (r = 0.43–0.48) between lower limb skin temperature and blood lactate during incremental exercise in sprinters and endurance athletes, which turns into a negative correlation during the recovery phase (r = −0.54∼-0.45). However, due to the low-frequency measurement of a single region (sampling interval ≥2 min during exercise, 5 min during recovery), the spatiotemporal regulatory mechanism of the interaction between the two remains unclear (Korman et al., 2024). This study fills the gap in previous research by synchronously collecting high-sampling-rate thermal imaging and blood lactate data, and directly verifies the strong correlation between chest temperature entropy and blood lactate concentration, as well as the existence of individual response differences.

This study demonstrates a strong correlation between chest temperature entropy and external load (watt) both at the overall and individual levels during incremental exercise, suggesting that the thermoregulation mechanism of the body may exhibit significant dynamic changes at different intensities of exercise. However, this study demonstrates the sensitivity of thermoregulation (entropy increase) in the chest region to both internal and external load increases. This region covers key respiratory muscles, including the heart, intercostal muscles, and external intercostal muscles (De Troyer et al., 2005), all of which increase metabolic activity during exercise, generating significant heat. Compared to the limbs, the chest has a thinner subcutaneous fat layer, which reduces thermal conductivity resistance, making it easier for deep tissue heat to be transferred to the skin surface (Rodríguez de Rivera et al., 2022; Neves et al., 2015). The surface thermal radiation pattern during exercise may be regulated by multiple systems. It is believed that IRT captures the Psr, which reflects the regulation of body surface blood vessels during exercise. Sympathetic nervous system activation, driven by the sympathetic-adrenal axis, releases norepinephrine and neuropeptide Y, which mediate the constriction of skin arterioles via α-adrenergic receptors, thereby regulating cutaneous blood circulation (Ootsuka and Tanaka, 2015). Meanwhile, increased muscle metabolic heat leads to elevated venous blood temperature. When venous blood temperature exceeds the core temperature threshold, the hypothalamus activates the vasodilation system through cholinergic non-adrenergic pathways, promoting the expansion of subcutaneous blood vessel networks (Demachi et al., 2013). At this point, heat is transferred from deep tissues to the skin surface through superficial veins and perforator vessels, forming a branching radiation pattern of the vascular tree to enhance skin thermal convection efficiency (Demachi et al., 2013; Hillen et al., 2020). This radiation pattern reflects the compensatory mechanism of the body to maintain core temperature stability by enhancing superficial blood flow heat dissipation under critical thermal load conditions. This study quantitatively characterizes the dynamic heterogeneity of chest skin thermal radiation patterns through entropy analysis and provides evidence for their significant synchronous dynamic correlation with blood lactate concentration, external load, and oxygen uptake during incremental exercise, as well as the existence of individual differences.

EPOC refers to the physiological phenomenon where oxygen consumption remains elevated above resting levels after exercise (Gaesser and Brooks, 1984). EPOC magnitude and duration vary according to exercise intensity and modality, with intermittent exercise generally producing a greater and more prolonged effect compared to continuous aerobic exercise (LaForgia et al., 2006). EPOC at different exercise intensities helps assess an individual’s recovery capacity and adaptability to exercise load. This study further supports the relationship between chest temperature entropy and EPOC. The overall correlation is weak, likely because thermoregulation during exercise is primarily controlled by blood circulation and heat dissipation mechanisms (Moreira et al., 2017). In contrast, the oxygen consumed during EPOC is mainly used to restore internal balance disrupted during exercise, including processes such as replenishing ATP and phosphocreatine, lactate clearance, and muscle glycogen resynthesis (Panissa et al., 2021). Moreover, the recovery of skin temperature lags behind the recovery of the energy metabolism system. During the post-exercise period, peripheral vasodilation persists after muscle contraction ceases, partly due to reduced venous return and sustained dilation of cutaneous vessels, contributing to post-exercise hypotension (Seeley et al., 2021). This vasodilatory response is mediated in part by cholinergic active vasodilation, which increases skin blood flow to dissipate heat generated during exercise (Francisco et al., 2023). In addition, nitric oxide released during recovery can directly relax cutaneous arterial smooth muscle, enhancing heat dissipation and potentially increasing local skin temperature in active muscle regions (Amato et al., 2025). These physiological processes may explain the observed elevation in chest temperature entropy during early recovery. Individual differences may be explained by the interaction of multiple factors, such as training experience, body composition, and local tissue metabolic states (Formenti et al., 2013; Priego Quesada et al., 2015; Weigert et al., 2018). Post-exercise metabolic rate may be related to cardiovascular fitness, mitochondrial and cellular remodeling, and autonomic nervous regulation efficiency (Greer et al., 2021). Although there is considerable individual heterogeneity and nonlinear associations between post-exercise thermal metabolic parameters and physiological recovery indicators, dynamic data from skin temperature fields obtained using infrared thermography, combined with entropy analysis of nonlinear dynamic features, can quantify the post-exercise thermal balance reconstruction process. This provides new biophysical markers for establishing a non-invasive, low-cost recovery monitoring system.

Based on the hierarchical clustering analysis results of incremental exhaustion exercise, chest temperature entropy demonstrates distinct stratified characteristics at different exercise intensities, effectively reflecting changes in metabolic states. The clustering analysis shows that chest temperature entropy has a good classification effect with the two exercise intensity markers, blood lactate and VO_2_. The three clusters correspond to low, moderate, and high physiological metabolic states at different exercise intensities. Chest temperature entropy can be used as an effective indicator for assessing exercise intensity, corresponding well with traditional physiological markers such as blood lactate and VO_2_. Compared to previous studies that used three-dimensional K-means clustering of VO_2_, entropy increase, and power into three clusters (Hu et al., 2025), this study further demonstrates the relationship between chest entropy increase and changes in internal load through hierarchical clustering, revealing the adaptability of using chest temperature entropy in exercise load assessment.

The strength of this study lies in the use of high-temporal-resolution infrared thermography to synchronously collect chest temperature entropy together with key exercise load monitoring indicators such as oxygen uptake, blood lactate concentration, and external power output during incremental exercise and recovery. This approach enabled a quantitative assessment of the association between thermoregulatory processes and metabolic responses, and the study provides the first empirical evidence of significant dynamic correlations between chest temperature entropy and traditional load monitoring indicators, supporting its potential feasibility as an exercise load assessment tool. Although this study demonstrates the potential of chest temperature entropy in monitoring metabolic responses during incremental exercise, it still has several limitations. Due to the need to expose the chest region for thermal imaging data collection, and for privacy protection and ethical considerations, this study included only 24 healthy young male participants. This design avoids the confounding effect of gender on temperature entropy but limits the generalizability of the findings to women or other age groups. Future work will explore ROIs that do not require direct chest exposure, enabling the safe inclusion of female participants and extending the applicability of this method to more diverse populations. Additionally, EPOC data were only collected during the first 10 min post-exercise, while excess post-exercise oxygen consumption can persist for several hours after high-intensity exercise. The lack of continued monitoring during the later recovery phase may have led to an underestimation of the dynamic correlation strength between temperature entropy and EPOC. Furthermore, due to the invasive nature of blood lactate monitoring, lactate concentration data were not collected during the recovery phase, preventing the exploration of the relationship between lactate metabolic recovery and changes in chest temperature entropy.

## 5 Perspective

The present findings suggest that infrared thermography–based chest temperature entropy analysis holds potential as a tool for monitoring exercise load. This method is non-invasive and relatively low-cost, enabling data acquisition without interfering with the activity itself and minimizing additional physiological or psychological stress on participants. As a complement to existing load monitoring systems, the temperature entropy parameter may offer an additional dimension of information related to thermoregulation and metabolic dynamics. Because this technique does not require wearable sensors, it could, in principle, be applied in open environments, in simultaneous monitoring of multiple individuals, and under non-laboratory conditions, offering practical applicability. Future studies with larger sample sizes and diverse activity contexts, combined with automated image extraction and computation techniques, are warranted to validate its stability and enhance its efficiency for real-world monitoring.

## 6 Conclusion

This study, using high-sampling-rate infrared thermography and simultaneous measurement of internal and external loads, is, to our knowledge, the first to reveal the quantitative relationship between chest temperature entropy and VO_2_, blood lactate concentration, and external load during exercise. It was found that the percentage increase in chest temperature entropy is significantly positively correlated in a non-linear fashion with these indicators, as well as positively correlated across individuals. Three-dimensional hierarchical clustering analysis shows that chest temperature entropy effectively distinguishes between low, moderate, and high metabolic load states and provides a good classification performance when compared to traditional physiological markers such as blood lactate and VO_2_. Furthermore, the relationship between chest temperature entropy and EPOC displays considerable individual heterogeneity. This study provides a quantitative surface thermal radiation pattern analysis through high-sampling-rate infrared thermography, offering a new biophysical marker for non-invasive monitoring of exercise load and recovery processes.

## Data Availability

The original contributions presented in the study are included in the article/Supplementary Material, further inquiries can be directed to the corresponding author.
